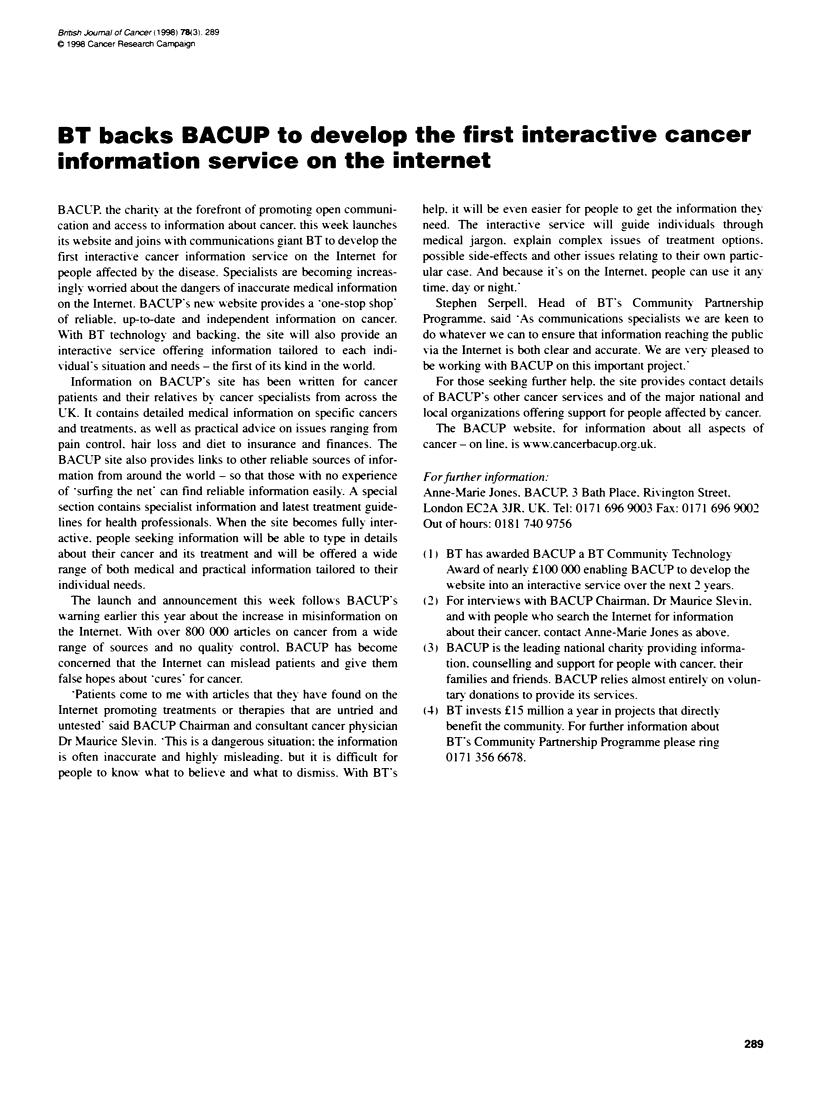# BT backs BACUP to develop the first interactive cancer information service on the internet

**Published:** 1998-08

**Authors:** 


British Journal of Cancer (1998) 78(3). 289
© 1998 Cancer Research Campaign

### BT backs BACUP to develop the first interactive cancer information service on the internet

BACUP. the charity at the forefront of promoting open communication and access to information about cancer. this week launches its website and joins with communications giant BT to develop the first interactive cancer information service on the Internet for people affected by the disease. Specialists are becoming increasingly worried about the dangers of inaccurate medical information on the Internet. BACUP's new website provides a 'one-stop shop' of reliable. up-to-date and independent information on cancer. With BT technology and backing. the site will also provide an interactive service offering information tailored to each individual's situation and needs – the first of its kind in the world.

Information on BACUP's site has been written for cancer patients and their relatives by cancer specialists from across the UK. It contains detailed medical information on specific cancers and treatments. as well as practical advice on issues ranging from pain control. hair loss and diet to insurance and finances. The BACUP site also provides links to other reliable sources of information from around the world – so that those with no experience of 'surfing the net' can find reliable information easily. A special section contains specialist information and latest treatment guidelines for health professionals. When the site becomes fully interactive. people seeking information will be able to type in details about their cancer and its treatment and will be offered a wide range of both medical and practical information tailored to their individual needs.

The launch and announcement this week follows BACUP's warning earlier this year about the increase in misinformation on the Internet. With over 800 000 articles on cancer from a wide range of sources and no quality control. BACUP has become concerned that the Internet can mislead patients and give them false hopes about 'cures' for cancer.

'Patients come to me with articles that they have found on the Internet promoting treatments or therapies that are untried and untested' said BACUP Chairman and consultant cancer physician Dr Maurice Slevin. 'This is a dangerous situation: the information is often inaccurate and highly misleading. but it is difficult for people to know what to believe and what to dismiss. With BT's help. it will be even easier for people to get the information they need. The interactive service will guide individuals through medical jargon. explain complex issues of treatment options. possible side-effects and other issues relating to their own particular case. And because it's on the Internet. people can use it any time. day or night.'

Stephen Serpell. Head of BT's Community Partnership Programme. said 'As communications specialists we are keen to do whatever we can to ensure that information reaching the public via the Internet is both clear and accurate. We are very pleased to be working with BACUP on this important project.'

For those seeking further help. the site provides contact details of BACUP's other cancer services and of the major national and local organizations offering support for people affected by cancer.

The BACUP website. for information about all aspects of cancer – on line. is www.cancerbacup.org.uk.

*For further information:*
Anne-Marie Jones. BACUP. 3 Bath Place. Rivington Street. London EC2A 3JR. UK. Tel: 0171 696 9003 Fax: 0171 696 9002
Out of hours: 0181 740 9756

(1) BT has awarded BACUP a BT Community Technology Award of nearly £100 000 enabling BACUP to develop the website into an interactive service over the next 2 years.
(2) For interviews with BACUP Chairman. Dr Maurice Slevin. and with people who search the Internet for information about their cancer. contact Anne-Marie Jones as above.
(3) BACUP is the leading national charity providing information. counselling and support for people with cancer. their families and friends. BACUP relies almost entirely on voluntary donations to provide its services.
(4) BT invests £15 million a year in projects that directly benefit the community. For further information about BT's Community Partnership Programme please ring 0171 356 6678.